# Hilbert-Huang versus Morlet wavelet transformation on mismatch negativity of children in uninterrupted sound paradigm

**DOI:** 10.1186/1753-4631-3-1

**Published:** 2009-02-02

**Authors:** Fengyu Cong, Tuomo Sipola, Tiina Huttunen-Scott, Xiaonan Xu, Tapani Ristaniemi, Heikki Lyytinen

**Affiliations:** 1 Department of Mathematical Information Technology, University of Jyväskylä, Jyväskylä, Finland; 2 Department of Psychology, University of Jyväskylä, Jyväskylä, Finland; 3 Hangzhou Applied Acoustic Institute, Hangzhou, PR China

## Abstract

**Background:**

Compared to the waveform or spectrum analysis of event-related potentials (ERPs), time-frequency representation (TFR) has the advantage of revealing the ERPs time and frequency domain information simultaneously. As the human brain could be modeled as a complicated nonlinear system, it is interesting from the view of psychological knowledge to study the performance of the nonlinear and linear time-frequency representation methods for ERP research. In this study Hilbert-Huang transformation (HHT) and Morlet wavelet transformation (MWT) were performed on mismatch negativity (MMN) of children. Participants were 102 children aged 8–16 years. MMN was elicited in a passive oddball paradigm with duration deviants. The stimuli consisted of an uninterrupted sound including two alternating 100 ms tones (600 and 800 Hz) with infrequent 50 ms or 30 ms 600 Hz deviant tones. In theory larger deviant should elicit larger MMN. This theoretical expectation is used as a criterion to test two TFR methods in this study. For statistical analysis MMN support to absence ratio (SAR) could be utilized to qualify TFR of MMN.

**Results:**

Compared to MWT, the TFR of MMN with HHT was much sharper, sparser, and clearer. Statistically, SAR showed significant difference between the MMNs elicited by two deviants with HHT but not with MWT, and the larger deviant elicited MMN with larger SAR.

**Conclusion:**

Support to absence ratio of Hilbert-Huang Transformation on mismatch negativity meets the theoretical expectations, i.e., the more deviant stimulus elicits larger MMN. However, Morlet wavelet transformation does not reveal that. Thus, HHT seems more appropriate in analyzing event-related potentials in the time-frequency domain. HHT appears to evaluate ERPs more accurately and provide theoretically valid information of the brain responses.

## Background

With the accumulation of knowledge concerning functioning of human brains, it has been found that millions of neurons self-organize into transient networks that synchronize in time and space to produce a mixture of short bursts of oscillations. These periodic vibrations can be observed and detected in the electroencephalogram (EEG) [1-3]. Event-related potentials (ERPs) are voltage fluctuations that are associated in time with some physical or mental occurrence. These potentials can be recorded from the human scalp and extracted from the ongoing EEG by means of filtering and averaging [4]. This article devoted to the study of an ERP through the averaged trace. We take mismatch negativity (MMN) as the example in this study. MMN is such a kind of negative ERPs, and it could be interpreted as the 'memory-mismatch' in [5] and 'regularity violation' in [6]. For example, MMN can be automatically elicited by the deviant stimulus in an oddball paradigm, in which the deviant stimulus occurs among repetitive and homogeneous stimuli. Hence, EEG recordings indeed are the superposition of different ERPs. If each ERP was regarded as an oscillatory phenomenon recordings would be considered as mixture of different oscillatory phenomena. MMN peaks about 150–200 ms after deviant onset with amplitude of peaks around -3 μV [1]. Compared to EEG MMN is very small, and to extract MMN efficiently is critical in MMN study.

**Figure 1:**
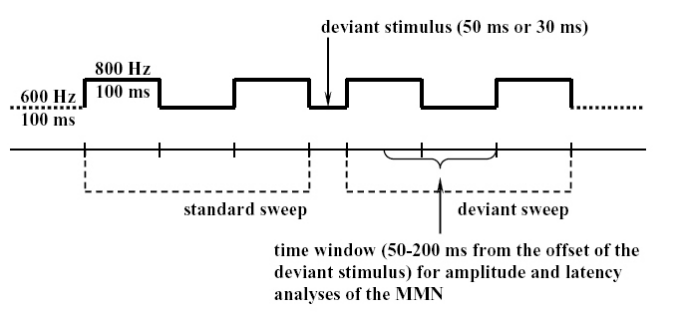
**Illustration of the MMN eliciting experimental paradigm**.

In order to evaluate the MMN-like oscillatory phenomenon from the ongoing EEG the averaging based difference wave (DW) [5], optimal digital filtering (ODF) [7] and wavelet decomposition [8] have been extensively used. Averaging over trials is a very popular algorithm to improve the signal to noise ratio. It assumes that in different trials the desired target signal is invariant, no artifacts are produced, and noises follow Gaussian distribution [9]. Under such assumptions the signal to noise ratio could be improved by an amount proportional to the square root of the number of trials [10]. In an oddball paradigm to elicit MMN DW is computed by subtracting the deviant sweep from the standard sweep (in Figure 1). In theory averaging removes the common exogenous processes from the standard and deviant sweeps. However, these assumptions do sometimes conflict with the reality. In addition to MMN, spontaneous ERPs can be generated and they may show in either the standard or deviant sweeps, which can produce noise when DW is subtracted [7]. Moreover, noises may not always follow Gaussian distributions. Hence, the performance of averaging can degenerate. Consequently, more data processing to the averaged trace is necessary. Band-pass digital filtering is an easily implemented method and can delete the frequency components out of a set frequency band. However, when ERPs overlap in the frequency domain such filters can not separate the overlapping components. Another problem is that the frequency resolution needs longer time series. This requirement can not be met when the desired ERP is transient. Wavelet filter [11] was developed to resolve this problem and has become very popular in many disciplines since the end of 1980s. It is very useful in analysing data with gradual frequency changes. Since wavelet transformation has an analytic form, it has attracted extensive attention of the applied mathematicians. Although wavelet filtering is versatile the disadvantage is that wavelet filter is not an adaptive method. The selection of the wavelet is too vital to make the method strict.

In addition to the drawbacks of these methods mentioned above they are directed either towards linear or stationary data or they assume deterministic processes. However, most actual data are nonlinear, nonstationary, and stochastic. In biomedical engineering, Klonowski [12] has declared that many biomedical researchers are 'infected with HLV – "Human Linearity Virus"'. They 'think linearly' and ignore the facts that human body and particularly human brain are complex nonlinear systems. These complex nonlinear systems generate non-stationary nonlinear signals and appropriate analysis of such signals does need new nonlinear methods.

Hilbert-Huang Transformation (HHT) is such a new adaptive method for analyzing nonlinear and nonstationary data. It was first defined by Huang and colleagues [13] in 1998. After its invention, HHT has been applied in many disciplines, such as analysis and correction of satellite data, data fusion from multi-sensors, speech analysis and speaker identification, machine health monitoring; biological, and physiological signals analysis, and so on [14]. HHT consists of two parts: (1) the empirical mode decomposition (EMD), and (2) the Hilbert spectral analysis. The key part of the method is the first step, the EMD, with which any complicated data set can be decomposed into a finite and often small number of intrinsic mode functions (IMF). An IMF is defined here as any function having the same number of zero-crossing and extrema, and also having symmetric envelopes defined by the local maxima, and minima. EMD is an adaptive method to identify the intrinsic oscillatory modes by the characteristic time scales in the data empirically, and to decompose the data accordingly. This decomposition is according to the straightforward extraction from the signal energy based on the various intrinsic time scales, and this technique adaptively decomposes non-stationary signals into a set of intrinsic time scales. In contrast to the previously discussed methods EMD is intuitive, direct, *a posterior *and adaptive, with the basis of the decomposition derived from the data. EMD has already been applied in the biomedical engineering [15-18].

In HHT the frequency defined as a function of time by differentiation rather than convolution analysis, and HHT is a more precise time-frequency representation method of signal than with "Fourier-type" methods [14]. Moreover, HHT does not impose *a prior *assumptions on the data as in Fourier methods (i.e., assumptions of linear and stationary data). Furthermore, EMD method is adaptive and therefore highly efficient. Since the decomposition is based on the local characteristic time scale of the data it is applicable to non-linear and non-stationary processes. With the Hilbert transform IMFs yield frequencies as functions of time that give sharp identifications of imbedded structures. Since MMN is a transient signal in the present study, HHT is applied to represent the time-frequency information of MMN. To reveal the effectiveness of HHT the popular Morlet wavelet transformation (MWT) [11] is also performed on the MMN, and the results of HHT and MWT are compared. MWT is a linear time-frequency representation procedure and applies the appropriate wavelet to decompose the signal into different levels and finds the proper levels to reconstruct the desired signal. MWT has advantages over traditional Fourier transforms in representing functions that have discontinuities and sharp peaks and for accurately decomposing and reconstructing finite, non-periodic, and non-stationary signals. However, MWT is not an adaptive method. If the wavelet model does not meet the signal, MWT may mislead the time-frequency representation of the signal. As MWT is a very popular method since 1990s it is not further described in this study. Please, see [11] for details.

## Method

### Experimental design and recordings

The same dataset as in [7] was utilized. Since the experiment has been described in detail elsewhere [7], only a brief introduction is provided in this article. The participants of MMN experiment were 102 children aged 8–16 years with normal hearing. Four of these participants were excluded due to data problem (mainly noisy). Thus, the total amount of participants included in analyses of this article was 98. The group of children with reading disability (RD) group consisted of 16 participants with mean age of 12 years and 2 months. The group of children with attention deficit (AD) included 16 participants with mean age of 11 years. The control group was formed of 66 participants with the mean age of 11 years and 11 months. The participants listened to alternating sound tones of 600 Hz and 800 Hz. The sound was continuous without pauses. Each repeated standard tone lasted 100 ms, whereas the deviant tones were either 50 ms or 30 ms in duration. Each deviant tone appeared in 7.5% of the trials. At least six repetitions of the standard 100 ms tones were presented between the otherwise randomly placed deviant tones [7]. The children listened to the stimulus sounds through earphones with an intensity of 65 dB. While listening participants watched a silent movie with subtitles and were instructed to sit still and disregard the sounds [7].

Nine channels were recorded with Electro-Cap International cap using the standard 10–20 sites. The channels included frontal (F3, Fz, F4), central (C3, Cz, C4) and parietal (Pz). In addition, mastoid channels (M1, M2) were recorded. To monitor eye movement, the upper left corner of the left eye (G1) and the lower right corner of the right eye (G2) were measured. Neuroline disposable electrodes were used for mastoid and eye movement measurements. The tip of the nose was used as the reference point for all electrodes. Impedances stayed at a level lower than 10 kOhm and mostly under 5 kOhm. Recordings were conducted with Brain Atlas amplifier (50 K gain) and Tecmar's Labmaster AD-converter using DSAMP software. During the recording the sampling rate was set to 200 Hz and the signal was processed with analog band-pass of 0 – 30 Hz [7].

### Data analysis

The averaged trace was computed for each participant at each electrode for each deviant. After this the first 300 ms of the averaged trace was utilized as the baseline. Hereinafter, the baseline of the averaged trace had been removed. Next, HHT was performed on each averaged trace. For the purpose of evaluating the performance of HHT MWT was also performed on the averaged trace. The half length of Morlet wavelet is 6. Next, the SAR was computed for the HHT and MWT. After this, SAR was averaged over channels. Finally, the MMN SARs were examined with the general linear model (GLM) and repeated measures of ANOVA. The variable was the SAR of the participants, and the factor was the deviant. This was done in order to test whether a difference would be observed between the MMNs elicited by the two deviants under two methods when data was treated with the two methods. Such a model has been adopted in [7,19-22] for similar analysis of MMN.

### Hilbert-Huang transformation

#### Empirical mode decomposition

HHT includes EMD and Hilbert transformation. EMD extracts the IMFs from the signal. An IMF needs to satisfy two criteria: First, the number of extrema and the number of zero crossings can differ by one at most. Second, the mean of the upper and the lower envelopes must equal to zero. These restrictions are necessary to meet the strict conditions for calculating instantaneous frequency. A signal is decomposed into these functions with sifting. The local maxima are connected with a cubic spline to form an envelope, after which the same is done to the local minima. Next, the mean of these envelopes *m*_1 _is calculated, and the first component *h*_1_(*t*) is obtained by subtracting the mean from the original signal through

(1) *x*(*t*) - *m*_1 _= *h*_1_(*t*).

This subtraction of the envelope mean from the component in question is repeated *k *times until a predefined condition of standard deviant between consecutive components is met. For different IMFs, the subtraction may be repeated different *k *times. Deviant limit is usually set between 0.2 and 0.3 [13]. This process is described as

(2) *h*_1_(*k *- 1, *t*) - *m*_1_(*k*) = *h*_1_(*k*, *t*),

(3) $$SD=\sum_{t=0}^{T}\frac{{\left|h_{1}\left(k-1,t\right)-h_{1}\left(k,t\right)\right|}^{2}}{h_{1}^{2}\left(k-1,t\right)}<0.3.$$

After the last subtraction the first IMF *c*_1_(t) is obtained,

(4) *c*_1_(*t*) = *h*_1_(*k*, *t*).

This is the IMF for further analysis, especially for calculating instantaneous frequency. The first IMF is subtracted from the original signal to obtain the first residue,

(5) *x*(*t*) - *c*_1_(*t*) = *r*_1_(*t*).

Consequently, another round of sifting is started using this residue as the signal. The first sifting process has extracted the highest frequency oscillation. The following siftings will produce the second highest oscillation etc.

The process is finished when *r*_*n*_(*t*) becomes monotonic or *c*_*n*_(*t*) or *r*_*n*_(*t*) has too small effect. After *n *rounds of sifting a residue is left

(6) *r*_*n*-1_(*t*)-*c*_*n *_= *r*_*n*_(*t*).

*r*_*n*_(*t*) is constant or represents a trend, and it is usually disregarded in the further analysis.

As a result, the original signal can be composed by summing up the IMFs and the last residue,

(7) $$x\left(t\right)=\sum_{i=1}^{n}c_{i}\left(t\right)+r_{n}\left(t\right).$$

#### Hilbert transform

Each of the IMFs is transformed into a complex plane with Hilbert Transform. The analytic signal *z*_*i*_(*t*) associated with IMFs *c*_*i*_(*t*) can be obtained as below,

(8) $$z_{i}\left(t\right)=c_{i}\left(t\right)+j\cdot H\left[c_{i}\left(t\right)\right]=a_{i}\left(t\right)e^{j\cdot \theta_{i}\left(t\right)},$$

(9-1) $$H\left[c_{i}\left(t\right)\right]=\frac{1}{\pi }\text{P}\int_{-\infty }^{\infty }\frac{c_{i}\left(u\right)}{t-u}du,$$

(9-2) $$a_{i}\left(t\right)=\sqrt{c_{i}^{2}\left(t\right)+H^{2}\left[c_{i}\left(t\right)\right]},$$

(9-3) $$\theta_{i}\left(t\right)=\arctan\left(\frac{H\left[c_{i}\left(t\right)\right]}{c_{i}\left(t\right)}\right),$$

where, $\sqrt{-1}=j$, *a*_*i*_(*t*) and *θ*_*i*_(*t*) represent the instantaneous amplitude and phase respectively. P indicates the Cauchy principal value. The instantaneous amplitude *a*_*i*_(*t*) is the distance of the values from the time axis in the complex coordinate analytic signal. The instantaneous phase *θ*_*i*_(*t*) is acquired from the angle in the complex plane. The IMFs have a narrow band characteristic that is required for the instantaneous frequency to be meaningful. The instantaneous frequency *ω*_*i*_(*t*) is calculated as the derivative of the phase,

(10) $$\omega_{i}\left(t\right)=\frac{d\theta_{i}\left(t\right)}{dt}.$$

Hence, at a given time *t*, the time-frequency analysis of *x*(*t*) through HHT could be expressed by

(11) *F*(*f*_*i*_, *t*) = |*a*_*i*_(*t*)|,

(12) $$f_{i}=\frac{1}{2\pi }\omega_{i}\left(t\right).$$

### Support to absence ratio

After the time-frequency analysis, a data matrix with dimensions of time by frequency is generated. In this study, the frequency range should correspond to the spectral feature. The time range of a trace when the desired ERP is present will be called signal's support, whereas other parts of the trace are called signal's absence [9]. We define the support to absence ratio (SAR) on the time-frequency plane as below,

(13) $$SAR=20\log10(\frac{TF_{s}}{TF_{A}}),$$

(14) $$TF_{s}=\frac{1}{T_{s}}\sum_{f=F_{L}}^{F_{H}}\sum_{t=T_{1}}^{T_{2}}\left|F\left(f,t\right)\right|,$$

(15) $$TF_{A}=\frac{1}{T-T_{s}}\sum_{f=F_{L}}^{F_{H}}\left[\sum_{t=1}^{T}\left|F\left(f,t\right)\right|-\sum_{t=T_{1}}^{T_{2}}\left|F\left(f,t\right)\right|\right],$$

where *F*(*f*, *t*) is the time-frequency analysis of a trial's trace, *T *is the length of the trace, [*F*_*L*_, *F*_*H*_] is the ERP optimal frequency band, [*T*_1_, *T*_2_] is the time interval of the ERP. SAR can reflect the characteristics of support signal based on its timing and spectral features.

It should be noted that according to the equation (13), a bigger SAR should correspond to a more evident support signal within its time-frequency representation.

In our MMN experiment the sampling frequency was 200 Hz, and the optimal frequency band was 2–8.5 Hz [7]. Each trial lasted 650 ms. As shown in Figure 1, the MMN time window of the 50 ms deviant started from 400 ms to 550 ms, whereas the MMN time window of the 30 ms deviant lasted from 380 ms to 530 ms.

## Results

Since the original dataset was the same as the one used in [7], the basic MMN peak and latency results were reported in [7], and are not discussed here. One important result should be stated though: The children did show MMNs, and the MMN peak amplitude was statistically larger than the baseline [7]. This study was focused on the main effect of SAR with HHT and MWT applied to the MMNs elicited by the two deviants. Thus, SAR was averaged over channels. The results consisted of 98 participants by two deviants under HHT and MWT respectively.

**Figure 2:**
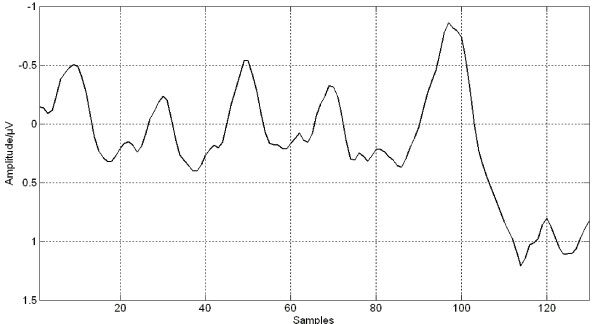
**Grand averaged trace at Fz under 50 ms deviant**.

**Figure 3:**
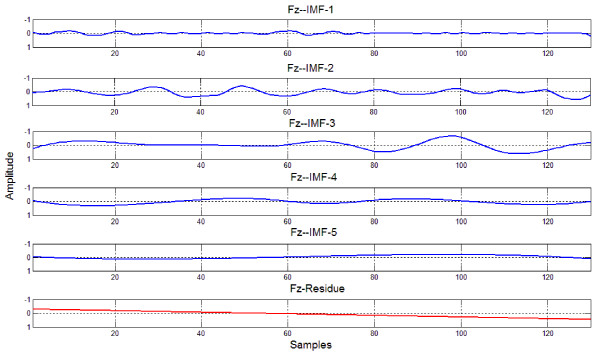
**IMFs of the trace in Figure **2.

**Figure 4:**
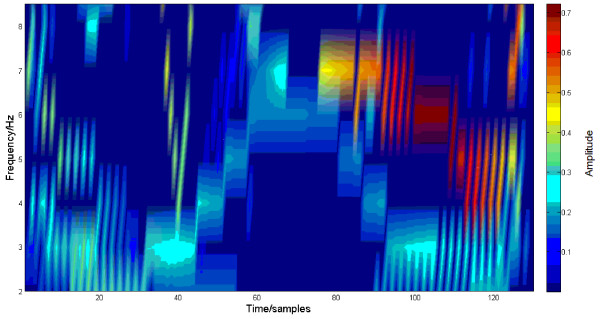
**HHT on the trace in Figure **2.

**Figure 5:**
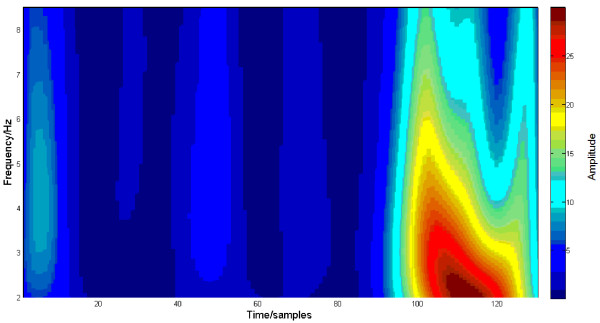
**MWT on trace in Figure **2.

The MMN peak amplitude was the largest at Fz channel [5]. Thus, the grand average trace of MMN elicited by the 50 ms deviant over all participants and trials at Fz channel, its IMFs and time-frequency representation with HHT and with MWT are demonstrated in Figure 2, Figure 3, Figure 4 and Figure 5 respectively. Five IMFs were generated by EMD. From the first IMF to the fifth IMF, the frequency of IMF turns lower. The MMN frequency range was set between 2 Hz and 8.5 Hz according to [7].

The SAR of all participants was statistically tested with HHT and MWT. The factor was the deviant type. For HHT main effect of LEVEL: F(1,97) = 8.87, P < 0.004; For MWT main effect of LEVEL: F(1,97) = 3.37, P < 0.074. Moreover, for HHT the averaged SAR over all participants MMN elicited by the 30 ms deviant was 7.28 times larger than the MMN elicited by the 50 ms deviant. With MWT this ratio was 1.03.

## Discussions

The classic ERP single-channel data-analysis methods include averaging, digital filtering and sometimes wavelet transformation. However, these methods are linear, and the brain is a nonlinear system. The contribution of this article was to present an appropriate nonlinear procedure–Hilbert Huang Transformation (HHT) to evaluate the mismatch negativity (MMN) and to show the effectiveness of HHT with comparison to Morlet wavelet transformation (MWT).

Theoritically, in the ERP data elicited in an oddball paradigm each trial should contain response to the repeated stimuli or deviant stimulus, other ERPs and background noises. Figure 3 demonstrated that the original trace in Figure 2 was decomposed into five IMFs, and each IMF represents an oscillator.

Apparently, the third IMF is the MMN-like trace, i.e., EMD decreases the noises. In Figure 4, HHT shows much clearer, sharper and sparser time-frequency representation of MMN than MWT. MWT is a linear and Fourier based time-frequency transformation. Furthermore, the frequency is defined through convolution of the whole signal and a wavelet. However, the HHT is a nonlinear method and the frequency is based on two adjacent samples, and this helps to separate overlapped ERPs. Particularly, the N1 and P3a will overlap MMN in some oddball paradigms [5]. HHT would reduce contamination to MMN by them. Thus MWT changes more gradually with time and frequency and due to this time-frequency distribution reached with MWT is smoother and wider, while, the representation of MMN with HHT should become much closer to the true evaluation of the MMN timing and frequency characteristics.

Statistical results of all participants show the larger duration deviant to elicit larger SAR with HHT. This meets the theoretical expectations of MMN elicitation [5]: Larger deviants should elicit MMNs with bigger peak amplitude and shorter peak latency. In addition, the time-frequency SAR contains both time and frequency information of MMN, whereas MMN peak amplitude only provides the information of MMN in the time domain.

This study is based on the average of all the trials for each participant. MWT is a linear transformation, obeying the rule of linear superposition. As we stated in the section of the introduction, more trials may improve the signal to noise ratio. As a result, to make the MWT perform the best in our experiment paradigm, all trials have been utilized for the averaging. However, the averaging is a linear operation, and could weaken the nonlinearity of the raw EEG data. For example, the IMFs' number of HHT reflects the complexity of the data series. Since the performance of HHT should degenerate through averaging, we do not analyze the IMFs' number in this presentation. But it will be very interesting to study the data's complexity under HHT on the single trial. In this current study, the shortcoming of MMN data to HHT is the limitation of the data length. Only 130 data samples are subjected to HHT. As the sampling frequency is 200 Hz, the frequency discrimination is too coarse. If the single trials are connected, this limitation will be released. Hence, for the entire decomposition of the data, it is promising to study the HHT in the analysis of the concatenated single trials of MMN. Moreover, it is desired to use fewer trials in the clinical study of MMN, and on the contrary, averaging based methods require more trials for higher signal to noise ratio. However, to the HHT on the concatenated trials, this confliction would not exist. This is because the experiment paradigm does not change over trials and it will be redundant to concatenate hundreds of trials together. The requirement of such a procedure is that the concatenated single trials could cover all key brain responses to the elicitation. This issue will be further studied in the future.

## Conclusion

As expected by the theory, mismatch negativity (MMN) elicited by larger deviant has larger support to absence ratio (SAR) of MMN when applying Hilbert-Huang transformation (HHT); however, the Morlet wavelet transformation (MWT) does not produce such results. This validates the nonlinear time-frequency representation method, HHT, as an appropriate tool for analyzing MMN. Compared to the linear TFR method, MWT, HHT is more sensitive. As the application of HHT on MMN is just an example for the study of HHT on ERPs, and there is no special requirement to facilitate the data processing procedure, this attempt suggests that HHT would be useful to study other ERPs.

## Competing interests

The authors declare that they have no competing interests.

## Authors' contributions

FC put forward the idea of Hilbert Huang Transformation on mismatch negativity, computed the Morlet Wavelet Transformation, developed the algorithm of support to absence ratio, performed the statistical tests and analyses, and composed the article. TS computed the time-frequency representation of HHT, computed the SAR, and revised the article. THS discussed MMN with FC and helped to proofread English. XX taught HHT to FC and provided the demo of HHT. TR discussed the idea and article with FC and TS. HL provided information on mismatch negativity to FC.

## References

[B1] Lachaux JP, Rodriguez E, Martinerie J, Varela FJ (1999). Measuring phase synchrony in brain signals. Hum Brain Mapp.

[B2] Lopes da Silva F, Neidermeyer E, Lopes da Silva F (1987). EEG analysis: theory and practice.

[B3] Olbrich E, Achermann P (2004). Oscillatory events in the human sleep EEG-detection and properties. Neurocomputing.

[B4] Winkler I (2007). Interpreting the Mismatch Negativity. Journal of Psychophysiol.

[B5] Picton TW, Bentin S, Berg P (2000). Guidelines for using human event-related potentials to study cognition: Recording standards and publication criteria. Psychophysiology.

[B6] Näätänen R (1992). Attention and brain function.

[B7] Kalyakin I, González N, Joutsensalo J, Huttunen T, Kaartinen J, Lyytinen H (2007). Optimal digital filtering versus difference waves on the mismatch negativity in an uninterrupted sound paradigm. Dev Neuropsychol.

[B8] Cong F, Ristaniemi T, Lyytinen H (2008). ERP qualification exploiting waveform, spectral and time-frequency infomax. proceeding of 3rd International Symposium on Communications, Control, and Signal Processing, Malta, 12–14 March 2008.

[B9] Möcks J, Gasser T, Köhler W (1988). Basic statistical parameters of event-related potentials. Journal of Psychophysiol.

[B10] Harmony T, John ER, Thatcher RW (1984). Neurometric assessment of brain dysfunction in neurological patients.

[B11] Burrus CS, Gopinath RA, Guo H (1998). Introduction to Wavelets and Wavelet Transforms: A Primer.

[B12] Klonowski W, Alexei Katashev, Yuri Dekhtyar, Janis Spigulis (2008). Importance of nonlinear signal processing in biomedicine.

[B13] Huang NE, Shen Z, Long SR (1998). The empirical mode decomposition and the Hilbert spectrum for nonlinear and non-stationary time series analysis. Proc Roy Soc London.

[B14] Huang N (2003). Hilbert-Huang Transform-A method for analyzing nonlinear and nonstationary data.

[B15] Li X (2004). Temporal structure of neuronal population oscillations with empirical model decomposition. Phys Lett A.

[B16] Li X, Sleigh J, Voss L (2007). Measure of the electroencephalographic effects of sevoflurane using recurrence dynamics. Neurosci Lett.

[B17] Liang H, Bresser S, Desimone R (2005). Empirical mode decomposition: a method for analyzing neural data. Neurocomputing.

[B18] Liang H, Lin Z, McCallum RW (2000). Artifact reduction in electrogastrogram based on the empirical model decomposition method.

[B19] Huttunen T, Halonen A, Kaartinen J, Lyytinen H (2007). Does mismatch negativity show differences in reading disabled children as compared to normal children and children with attention deficit hyperactivities disorders?. Dev Neuropsychol.

[B20] Pakarinen S, Takegata R, Rinne T (2007). Measurement of extensive auditory discrimination profiles using the mismatch negativity (MMN) of the auditory event-related potential (ERP). Clin Neurophysiol.

[B21] Kalyakin I, González N, Kärkkäinen T (2008). Independent component analysis on the mismatch negativity in an uninterrupted sound paradigm. J Neurosci Meth.

[B22] Huttunen T, Kaartinen J, Tolvanen T (2008). Mismatch negativity (MMN) elicited by duration deviations in children with reading disorder, attention deficit or both. Int J Psychophysiol.

